# Challenges, Solutions, and Quality Metrics of Personal Genome Assembly in Advancing Precision Medicine

**DOI:** 10.3390/pharmaceutics8020015

**Published:** 2016-04-22

**Authors:** Wenming Xiao, Leihong Wu, Gokhan Yavas, Vahan Simonyan, Baitang Ning, Huixiao Hong

**Affiliations:** 1 National Center for Toxicological Research, U.S. Food and Drug Administration, 3900 NCTR Road, Jefferson, AR 72079, USA; Leihong.Wu@fda.hhs.gov (L.W.); Gokhan.Yavas@fda.hhs.gov (G.Y.); Baitang.Ning@fda.hhs.gov (B.N.); Huixiao.Hong@fda.hhs.gov (H.H.); 2 Center for Biologics Evaluation and Research, U.S. Food and Drug Administration, 10903 New Hampshire Ave, Silver Spring, MD 20993, USA; Vahan.Simonyan@fda.hhs.gov

**Keywords:** genome, sequencing, assembly, personal genome, quality metrics

## Abstract

Even though each of us shares more than 99% of the DNA sequences in our genome, there are millions of sequence codes or structure in small regions that differ between individuals, giving us different characteristics of appearance or responsiveness to medical treatments. Currently, genetic variants in diseased tissues, such as tumors, are uncovered by exploring the differences between the reference genome and the sequences detected in the diseased tissue. However, the public reference genome was derived with the DNA from multiple individuals. As a result of this, the reference genome is incomplete and may misrepresent the sequence variants of the general population. The more reliable solution is to compare sequences of diseased tissue with its own genome sequence derived from tissue in a normal state. As the price to sequence the human genome has dropped dramatically to around $1000, it shows a promising future of documenting the personal genome for every individual. However, *de novo* assembly of individual genomes at an affordable cost is still challenging. Thus, till now, only a few human genomes have been fully assembled. In this review, we introduce the history of human genome sequencing and the evolution of sequencing platforms, from Sanger sequencing to emerging “third generation sequencing” technologies. We present the currently available *de novo* assembly and post-assembly software packages for human genome assembly and their requirements for computational infrastructures. We recommend that a combined hybrid assembly with long and short reads would be a promising way to generate good quality human genome assemblies and specify parameters for the quality assessment of assembly outcomes. We provide a perspective view of the benefit of using personal genomes as references and suggestions for obtaining a quality personal genome. Finally, we discuss the usage of the personal genome in aiding vaccine design and development, monitoring host immune-response, tailoring drug therapy and detecting tumors. We believe the precision medicine would largely benefit from bioinformatics solutions, particularly for personal genome assembly.

## 1. Introduction

Following President Obama’s announcement of the new initiative for precision medicine, the NIH proposed a large scale sequencing project to sequence one million human genomes [1]. In order to promote the Precision Medicine Initiative’s mission and develop individualized patient level treatments [2,3], there is a strong need to interrogate the changes (e.g., mutations during cancer development) in genomes for each individual over the course of life span, known as *N-of-1* trials. More and more genetic mutations or defects are linked to various diseases [4], and database repositories are being created, providing storage and dissemination of such actionable mutations [5]. Identifying these variants in individual patients will be the key objective for an enhanced clinical diagnosis and prognosis. Therefore, this new practice is largely dependent on our ability to accurately document the background of one individual genome under its normal state.

Currently, all mutation and structural variant discovery are exclusively reliant upon the mapping of sequence reads to the reference genome, which was derived from the pooled DNA from several individuals [6]. Even though the reference genome has been improved over the past fifteen years, the latest build of the reference genome still has hundreds of gaps and unplaced scaffolds (see Table 1), owing to different haplotypes from original donors. Another pitfall of the current reference genome is that reference alleles of single nucleotide polymorphism (SNP) may actually represent minor alleles in the general population. It has been demonstrated that short sequence reads containing reference alleles of SNP have a higher preference to be mapped at a right position while reads with non-reference alleles of SNP will likely be misplaced [7]. Such bias can be worse if the reads contain a higher sequencing noise. In addition, studies have shown that the presence of SNP would have high impact on false positive and false negative rates of single nucleotide variant (SNV) calls, presumably, due to incorrect mapping of reads [8].

Furthermore, the 1000 Genomes Project and other studies have revealed that there is a high degree of copy number variation and structure variation among normal individuals [9,10]. A segment of genomic structure polymorphism ranges from 10 kb to 1 Mb in size and together these sum up to 12% of the genome in all combinations [9]. These segments harbor hundreds of genes and functional loci and display unique population characteristics [11]. Therefore, like the discovery of SNVs, using the reference genome for genomic structure variant discovery may result in higher rate of errors due to inconsistencies between actual and reference genomes.

The reference genome would be suitable for a large cohort study for genetic variant discovery at a global level so that variants with a high degree of recurrence within the study group would be easily identified. When linking genotypes to a certain phenotype on a single individual, the precise genome component for that individual would allow us to discover genetic defects that may reside within highly diversified sequence regions, such as olfactory receptor gene [12], Human leukocyte antigen (HLA) [13], drug metabolizing enzymes and transporters (DMETs) [14,15,16], and B-Cell/T-Cell receptor [17,18]. Moreover, certain genetic defects might be artifacts of the person’s genomic construct with copy number and structural variations, and not a result of mutations in any of the known genes [11]. Therefore, personal genomes are a significant step forward for a more comprehensive approach towards precision medicine [19,20].

Since the first draft of human genome was published in 2001 [21,22], the fast pace development of sequencing technology has resulted in entire genome sequencing at a tiny fraction of cost and time compared to the original human genome project (HGP) [23]. To date, over 200,000 individual human genomes have been sequenced [24], yet only two dozen have been assembled as personal genomes [25,26]. The significant lagging of assembled genomes compared to the number of individual whole genome sequencing (WGS) suggests many challenges in building personal genomes [27].

In this review, we provide some background information on how the first human reference genome was sequenced and assembled. We summarize the currently available Next Generation Sequencing (NGS) platforms and software packages for genome assembly and post processing. We also discuss the requirement of IT infrastructure for building personal genomes and currently available cloud-computing based services, and propose quality metrics and parameters for the assessment of an assembled genome. Finally, we consider the benefit of using personal genomes as references and approaches to be taken in order to obtain a reliable personal genome.

## 2. History of Human Genome Sequencing

The HGP was initiated in 1990 by the US government and expanded by five other countries (China, France, Germany, Japan, and the United Kingdom). This $3 billion project claimed to sequence euchromatic regions of the genome, which made up about 90% of the whole human genome, whereas the heterochromatic region is still the most challenging part in current genomic research field due to its high repetitive property. While this international consortium effort was underway, another effort was launched in 1998 by Celera Genomics Inc. Both groups declared a completed working draft in 2001 and published their assembled genomes in Nature and Science, respectively [21,22].

At the beginning of the human sequencing project, the general approach was to break the genome into fragments of ~150 kb and insert these fragments into a bacterial vector to create “bacterial artificial chromosomes” (BAC). Individual BAC clones were then picked and amplified by the bacterial DNA replication system in order to generate enough DNA materials for sequencing [28]. As the length of sequence from Sanger based sequencing machine was limited to a few thousands bases per run, a “chromosome walking” strategy was used to sequentially uncover DNA sequence of each BAC clone downstream from the start. This approach was extremely time consuming and labor-intensive. As “whole genome shotgun sequencing” was introduced by Roach *et al.* [29] in 1994, and was later on adopted by Celera Genomics, bioinformatics solutions started to play a critical role in the human genome sequencing project. Each “shotgun” fragment was less than 10 kb. Reassembling these “short” fragments into genome content largely rested on computational algorithms. Eventually, two approaches were married to form a new strategy, “hierarchical shotgun”. With this approach, “shotgun” fragments were derived from the BAC library and sequenced. While shotgun sequences could be assembled into relatively longer contigs (contiguous genetic sequence), the map of BAC clones would provide the skeleton for building chromosome scaffolds for final genome assembly [30]. The “hierarchical shotgun” was used to complete a current reference genome and is still being used for the further improvement of human reference genome.

Both assembled genomes from HGP and Celera Genomics are available from the National Center for Biotechnology Information (NCBI). While the HGP genome is used as the primary reference with complete package of annotation such as known genes/transcription mapping, SNPs, factor-binding motifs, sequence conservation, *etc.*, the Celera Genomics genome serves as the alternative reference. Besides the human reference genome, several individual genomes were also generated. James Watson and Craig Venter’s personal genomes have been published in 2007 with diploid information [31,32]. Just a few months later, an Asian individual genome (YH) was reported and compared to the previously available individual genomes [33]. More individual genomes (e.g., NA18507, Korean AK1, and NA12878) were generated and compared to the reference genome and alternative genomes [34,35,36].

Currently, various sequencing technologies available to sequence the human genome have promoted comprehensive research on individual genomes. For instance, Genome in a Bottle Consortium (GIAB) initiated by National Institute of Standards and Technology (NIST) in 2011 sequenced a few samples (one pilot genome NA12878 and two Personal Genome Project trios) with multiple technologies. The goals of the GIAB are not only to generate several individual personal genomes with well characterized genome sequence variations within a familial clade but also to provide standard reference samples for the community to calibrate sequencing platforms and computational software packages. Even though the project is still ongoing, a standard reference material (NA12878) and its associated genomic content are publically available [37].

## 3. Evolution of Sequencing Platforms

In 1975, Fred Sanger and his colleagues first introduced “sequencing-by-synthesis” [38]. The strategy relied on the random termination of DNA synthesis by incorporating dideoxynucleotide triphosphates (ddNTPs). Various sizes of synthesized DNA fragments labeled by radioactive isotopes were separated on a poly-acrylamide gel which was then exposed to a film. DNA sequence was derived by manually examining fragment positions on the film. While the whole process would take up to three days to finish, its throughput and accuracy were very minimal. In 1986, Leroy Hood revolutionized the sequencing technology by replacing isotope labeling with fluorescence whose signal would be detected by a machine during the process of DNA fragment separation [39]. The fluorescent-based machine, now called the “First Generation”, was further improved by fine separation of DNA fragments and parallel run, such as capillary-electrophoresis [40,41,42]. Compared to the manual sequencing method, the “First Generation” sequencing machine had improved throughput from a few hundreds bases to hundreds of thousands of bases. Run time and sequence quality were also improved significantly. The “Next Generation Sequencing” (NGS) technology started to emerge ten years ago, including 454, helicos, Solexa and SOLiD [43,44,45,46]. SOLiD was based on “sequence-by-ligation”, while the rest of technology used “sequencing-by-synthesis”. High throughput of sequences from NGS has significantly cut down the cost for DNA sequencing and consequently promoted activities for human whole genome sequencing [23,24].

While the “Next Generation” is still in its fast evolution phase, the “Third Generation”, represented by PacBio and Oxford Nanopore technology, has started to emerge. Here, we list sequencing platforms that are either active on the market or are expected to be coming soon. The performance and feature of each platform were summarized in Table 2.

### 3.1. Illumina Platforms

Illumina HiSeq2000 is one of the most widely used sequencing instruments launched by Illumina in 2010. This platform was derived from the Solexa technology by using a reversible termination blocking group and removable fluorescent labels so that DNA synthesis can be continued after one cycle of sequence detection. As the technology places millions and millions of sequence templates on a solid face, it can generate millions and millions of reads in parallel. Sequencing can be initiated from both directions of the template so that its “pair-ended” reads can potentially extend to biological applications such as the detection of fusion genes. HiSeq2000 also combines the optical systems and manufacturing processes, using two laser sources on the Flow Cell Scan with four cameras to further reduce signal interference. With dual flow cell, HiSeq2000 could have an output of almost 600 GB of 2 × 100 bp data in 11 days. Illumina’s HiSeq2500 is an upgrade version of HiSeq2000, which introduced a new, rapid run mode that allows researchers to generate 120 GB of data in 27 h. Recently, HiSeq X ten was developed for large-scale sequencing projects and claimed to deliver over 18,000 human genomes per year at the price of $1000 per genome, at a considerable coverage of 30×.

### 3.2. Roche 454

The 454 sequencing technology, purchased by Roche Diagnostics in 2007, was used for one of the first individual human DNA sequence projects after the HGP [31], and was also the first marketed NGS platform. The 454 platform is based on pyrosequencing technology, which relies on the detection of pyrophosphate released from nucleotide incorporation, rather than chain termination. Two major models, Genome Sequencer (GS) FLX system and GS Junior were designed for different research purposes. The GS FLX system using Titanium XL+ with Titanium series reagents could have an output of more than one million reads of 400 bp each in one run, whereas GS Junior offers about one tenth of the output of the FLX also with much less cost for the modest sequencing needs. The major advantage of the 454 platform was based on its relative long-reads. However, this advantage became trivial due to its high cost, especially when Illumina and Ion Proton have upgraded their technologies with comparable length of reads (e.g., from 25 to 150 bp).

### 3.3. Life Technology Ion Torrent

Ion Torrent, as its name describes, differs from other platforms according to its unique detection method of measuring the hydrogen ions that were released during the polymerization. Ion Proton is the latest technology of Ion Torrent, acquired by Life Technology in 2010, which could generate more reads than the Ion Personal Genome Machine (PGM). Its first generation chip (Ion Proton I) could generate ~10 Gb per run while its second chip (Ion Proton II) could generate ~30 Gb data per run in 2–4 h.

### 3.4. Qiagen Intelligent Biosystems

Intelligent Biosystem was purchased by Qiagen in 2012, with its two main platforms as Max-Seq and Mini-20. Max-Seq, its first released instrument, also used sequencing by synthesis technology and could generate up to 132 GB of high quality paired-end data with 35–55 bp reads in one run. Since Qiagen has changed their direction in the sequencing market, its next instrument Mini-20 has a much smaller throughput but also a lower cost that generates 80 GB reads of up to 2 × 100 bp per run, which would focus on the diagnostic and clinical markets.

### 3.5. Pacific Biosciences

The PacBio RS system uses single molecule real-time (SMRT) sequencing technology that is not dependent on DNA amplification. Using biologically engineered DNA polymerase and nucleotides labeled through phosphor-linkage, it reads sequences while the correct nucleotide is being incorporated during the DNA synthesis process [47]. It is the so-called “Third Generation Sequencing” platform and generates a relatively small number of rather long reads (>10 kb) instead of a large number of short reads (<200 bp). The current read length of PacBio RS II system was up to 60 kb length reads with most of the higher quality reads being around 10–20 kb [48]. Although with a higher read error compared to other platforms, its error model is stochastic that is significantly improved by circular consensus sequencing. Because of the long reads, PacBio sequencing has been applied to model organisms and produced significantly better results, especially on *de novo* assembly where the longer reads provide a local frame for shorter read assembly [49,50,51].

### 3.6. Oxford Nanopore

Oxford nanopore technology (ONT) is a very unique sequencing technology that is independent of any kind of DNA enzyme activities [52,53]. ONT reads a DNA strand directly when it passes through a nanopore formed by biologically modified alpha-hemolysin membrane protein. The blockage of ion flow through the nanopore by passing-by nucleotides varies depending on their types. Thus, by monitoring the change of ion current while a single DNA strand passes through a nanopore, the sequence can be inferred [54]. MiniON, an early product from ONT, can produce read up to 200 kb in length [55,56]. However, low throughputs and higher sequencing error rate (15%–20%) are limiting its usage in genome assembly [56].

## 4. Current Solutions for *De Novo* Assembly and Post-Assembly

As mentioned in the introduction section, due to the pitfalls of reference genome and mapping biases in alignment approach, full discovery of genetic variations of an individual merely relies on *de novo* assembly of the personal genome [57]. Hence, in this section, we review current solutions for *de novo* assembly and post-assembly process.

### 4.1. De Novo Assembly Approaches

There are two major approaches for *de novo* genome assembly: overlap/layout/consensus (OLC) and *de Bruijn* graph. OLC consists of three major steps: (1) all overlaps among reads are first identified; (2) a layout (L) of all the reads and their overlaps information is then constructed as a graph and (3) the consensus (C) sequence is finally inferred from the graph. Software packages such as SSAKE, SHARCGS, VCAKE, Celera Assembler, Arachne, and PCAP take the OLC approach [58,59,60,61,62,63].

A *de Bruijn* graph assembly is based on *k*-mer graphs from the input reads. The nodes of the graph are constituted with *k*-mers, the seed sequence (shorter than the length of reads). The edges of each node represent two adjacent *k*-mers overlapped with the length of *k*-1 in a reads. All possible combinations of these types of graphs are then searched for within the entire input reads for exact matches or matches after error correction. A graph with multiple traverses represents the repeat structure of the genome; whereas a graph with single path depicts a non-repetitive region. With all *k*-mer graphs stored as a hash table in computer memory, searching on real reads or read pairs would provide evidence for path extension. In general, a *de Bruijn* graph is suitable for small genomes with less complexity in content than diploid genome sequence and repeated regions longer than *k*-mer would compromise assembly outcomes [64]. A *de Bruijn* graph is applied in tools such as EULER, ALLPATHS, Velvet, ABySS and SOAPdenovo2 [65,66,67,68,69].

While OLC works better for relatively longer reads (100–800 bp), a *de Bruijn* graph is more suitable for short reads (25–100 bp) and requires much higher computational memory [70]. More detailed description and comparison between OLC and *de Bruijn* could be found in Li’s review paper [71]. There are more than 50 software packages available today for genome assembly. A crowdsourcing effort by the Assemblathon consortium has gone through two phases to evaluate the performances of dozens of assemblers [72,73]. Results from Assemblathon and other studies indicated that there is no “one-size-fits-all” algorithm for *de novo* assembly [64,74,75]. Performance of assembler would largely depend on sophisticated properties of genome content, and NGS data such as error rate, read depth, *etc.* [64]. Even though no particular package stands out as the best solution for genome assembly, some key measurements for overall quality of an assembly have been established [73]. While it is an important endeavor, the Assemblathon studies only focused on simulated (artificial) data or data from non-mammalian vertebrate species [72,73]. To our knowledge, no similar study has been conducted on whole human genome, which is much larger in size and more complicated in content.

### 4.2. Post-Assembly Approach

As *de novo* assembly might create poor-quality drafts, primarily due to short scaffolds, false joining of contigs, or errors at base level, a post-assembly approach is designed to improve the quality through the following major functions:

(1) *Contigs orientation and visualization.* Tools such as CONTIGuator, Projector2, OSLay, and r2cat could are to visualize and arrange contigs and estimate gaps compared with a reference genome [76,77,78,79].
(2) *Extending contigs and filling gaps.* Tools such as GAA program and Reconciliator, integrate several different generated assemblies to extend or merge contigs [80].
(3) *Reads error correction.* Tools such as ICORN, Artemis and AutoEditor are used to improve base calling accuracy, correct indels and deal with repeated regions [81].
(4) *Unmapped reads Annotation.* Tools such as Ensembl, GARSA, and SABIA have also been widely used for improving genome annotation [82,83,84].

Additionally, a comprehensive post-assembly approach has been developed recently. For instance PAGIT (post-assembly genome-improvement toolkit) [85], incorporates four open-access post assembly algorithms, ABACAS (algorithm-based automatic continuation of assembled sequences), IMAGE (iterative mapping and assembly for gap elimination), ICORN (iterative correction of reference nucleotides), and RATT (rapid annotation transfer tool). ABACAS is a contig ordering and orientation tool which orients contigs and estimates the gaps between contigs in comparison with a reference sequence [86]. IMAGE is an approach which uses the properties of paired-end reads of Illumina to close gaps and extend contigs [87]. ICORN is designed to correct small sequencing errors such as those from low-quality bases [81], and finally, RATT is used to annotate the new assembly with high quality references from closely related species [88].

Frequently, the outcomes of the assembly programs are provided in the form of highest scoring contigs and do not properly demonstrate the alternatives from the pool of all available graphical trajectories mentioned above. Consumers of the contig information are left with a false sense of reliability of the proposed scaffold without a hint to what was the next best potential contig in the candidate list, which might have ultimately turned out to be a better choice from biological sense or as a meta-assembly for all chromosomes of the species.

Furthermore, approaches based on multiple assemblies could also improve the final genome contiguity and quality. For instance, Wences *et al.* developed Metassembler, which could merge multiple assemblies into a single superior sequence [89]. Deng *et al.* developed an ensemble strategy to integrate various assemblers with a novel partitioned sub-assembly approach to generate better contigs [90]. While they all attempt to improve the continuity, completeness, and accuracy of assemblies, each software package has its own unique advantages and disadvantages. Therefore, multiple iterations of post assembly processing are necessary to warrant a comprehensive genome assembly.

Above all, the approach of genome assembly has developed rapidly and could gradually handle larger genomes. The brief description of common assembly and post-assembly approaches are listed in Table 3. Although there are still large inconsistencies between different assemblers, combining long reads and short reads for hybrid assembly and also post-assembly approaches have shown their bright prospects in genome improvement. Moreover, developing *de novo* assembly management system could also help researchers to handle various types of assembly results [91].

## 5. Coming Era of Long-Reads Sequencing and Hybrid Assembly

**Figure 1:**
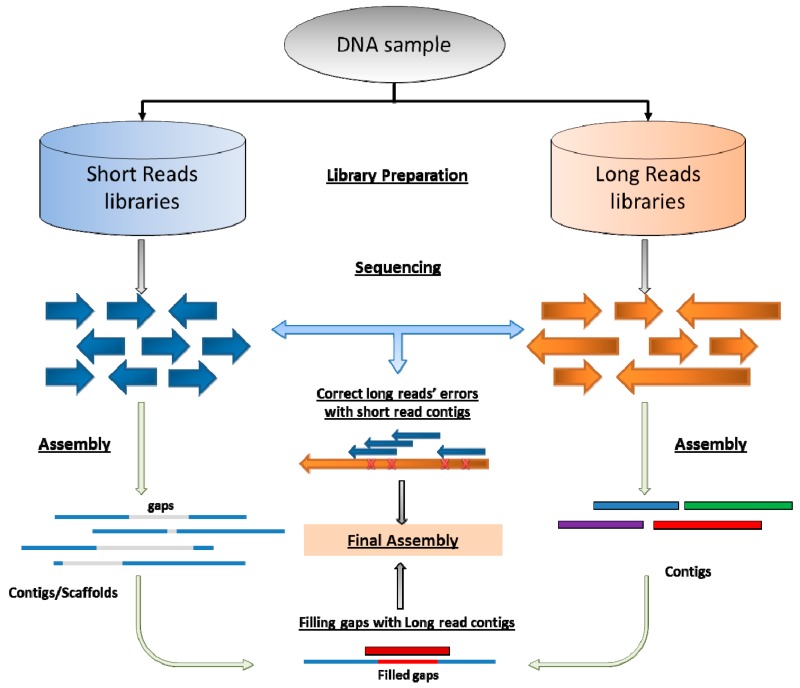
Common flowchart of hybrid assembly to integrate short and long reads. The combination can be at the reads level, *i.e.*, using short reads to correct the errors in long reads. Alternatively, long reads or their derived contigs could be used as bridges to join or fill-in gaps of contigs assembled with short reads.

In the last five years, long read sequencing has rapidly developed, granting long read assembly algorithms more attention in dealing with new types of genomic data. Long reads sequencing platforms, such as PacBio, have specific *de novo* assembly algorithm (e.g., Hierarchical Genome Assembly Process [HGAP] [51], and FALCON toolkit [92]) based on the OLC algorithm. The assembly result of long reads would significantly contribute to the personal genome (Figure 1). For instance, Pendleton *et al.* combined single-molecule sequencing (PacBio) with single-molecule genome maps (BioNano Irys) for human *de novo* assembly. This hybrid assembly markedly improves upon the contiguity observed from traditional shotgun sequencing approaches, with scaffold N50 values approaching 30 Mb, and identified complex structural variants (SVs) missed by other high-throughput approaches [93].

As illustrated in Figure 1, long-read sequencing could be combined with short-reads data, in order to improve the assembly quality and fill the gaps generated by conventional NGS platforms. For instance, combining PacBio data with Illumina and Roche 454 data could significantly improve the N50 length. Using short, high-fidelity sequences to correct the sequencing errors in single-molecule sequences from PacBio RS platform could lead to a more accurate assembly result [56]. The combination of Illumina and PacBio sequence data assembled through the ALLPATHS-LG algorithm gave the best summary statistics and most accurate rDNA operon number predictions, for four different bacteria [94]. Peng *et al.* combined multiple sequencing platforms, including 454 FLX, Illumina HiSeq 2000 and PacBio for *de novo* assembly of Horseweed (*Conyza canadensis*). The assembly covered 92% of the genome, including the complete chloroplast genome and a nearly complete mitochondrial genome [95].

Long reads sequencing could also be used as the intermediate bridge for short reads assembly. Laszlo *et al.* reported Nanopore sequencing reads could be used to align short reads such as Illumina MiSeq to facilitate rapid and accurate sorting of short sequence reads in proper order [96]. Pendleton *et al.* combined short and long reads for phasing single-nucleotide and structural variants, which generate haplotypes with high consistency in trio-based studies [93]. In addition, using hybrid sequencing of short and long reads could rapidly find disease-associated long STRs in personal genomes [97].

## 6. Computer Infrastructure Needs for Genome Assembly

### 6.1. Data Storage

NGS platforms usually generate massive amounts of raw data that normally requires very large storage. The amount of sequencing data produced by an NGS platform for a sample mostly depends on the type (*i.e.*, exome, whole genome, targeted, and *etc.*) and depth of coverage of the sequencing experiment and the size of the sequenced genome. As a DNA sample is sequenced at a higher depth of coverage, the size of the raw data produced out of this sequencing experiment will be larger. As an example, 45 GB of sequencing data (in zipped format) was produced in three Illumina HiSeq2500 runs with a total coverage of 32× for HapMap sample NA12878.

On the other hand, the amount of disk space required to store the assembled genomes is much smaller compared to the raw sequencing data. For instance, the compressed human genome build GRCh38/hg38 is only 938 Mb.

Based on the above examples, the space needed to store and transmit a single raw NGS dataset may seem feasible, but it becomes challenging to store, manage and transmit these datasets when the number of data samples goes up to hundreds, or even thousands and the coverage of the sequencing experiments goes deeper. Currently, many of the bioinformatics research facilities maintain their own High Performance Computing (HPC) cluster systems, which are basically a set of connected computers (*i.e.*, computing nodes) via a fast local area network that perform scheduled tasks in parallel and is controlled by centralized management software. Different distributions of GNU/Linux are commonly used as the underlying operating system of the HPCs. The data is generally shared through a clustered file system, which allows the nodes of the HPC system to simultaneously access the NGS data stored on shared disks. This computing paradigm poses some challenges for the efficient analysis of NGS data in general:

(1) The HPC clusters require a dedicated facility along with computer hardware and IT personnel, which is expensive. Additionally, ever growing number of associated data types require databases to store and manipulate metadata efficiently. This creates a need for database administration, which is an added cost.
(2) In the near future, the scope of sequencing projects is expected to grow due to the reduced sequencing costs; hence, more samples will be sequenced within each project. In that case, the NGS data will become overwhelming and it will not be possible to efficiently store the data in a limited number of disks. Moreover, the need to keep backup copies of the data sets, which should be maintained to prevent accidental data loss, imposes extra data management and storage costs. Given the fact that enterprise level NGS platforms currently hold petabytes of data, the cost of the high fidelity backup can very expensive.
(3) Currently, transferring the data from the NGS platform to a file system where the data is analyzed is carried out by either copying it into a large storage device then shipping it to the destination or by transmitting it over the Internet, which is bound by the network bandwidth. The network speeds are too slow to allow the transfer of terabytes of NGS data routinely over the Internet. With the advent of Internet 2 [98], there is significant optimism in the market; however, the production of new NGS datasets outpaces the growth in network throughput.

### 6.2. CPU/Memory

The *de novo* genome assembly is a demanding task in terms of CPU and memory requirements. In the Assemblathon phase II study, three different species’ genomes, sequenced by three different platforms, namely Illumina Hiseq2000, Roche 454 and PacBio, were assembled by various *de novo* assembly pipelines. The estimated genome sizes for these species are between 1 and 1.6 Gb [73]. A total of 43 assemblies from 21 participating teams were evaluated utilizing a combination of optical map data, fosmid sequences, and several statistical methods. The teams used various assembly pipelines such as ABySS, ALLPATHs-LG, SOAPdenovo, and *etc.*, to assemble the genomes for these three species. However, these pipelines greatly differ in terms of their CPU and memory requirements. The minimum and maximum amounts of required memory were reported to be 64 GB (the CoBiG2 team using 4Pipe4 pipeline, Seqclean, Mira, and Bambus2 as the primary assembly software used) and 1.2 TB (the MLK Group using the ABySS) [69], respectively. Furthermore, almost all of the tools require extensive computational power, such as an HPC with more than 20 nodes with several cores on an average. Even with these extensive CPU/memory requirements, the assemblers require at least several days to compute the assembly. Not only is it expensive to execute this in a clinical setting, but the outcomes are also frequently not-deterministic, meaning that the repeat execution under different CPU/memory configuration may result in a different outcome because of the inherent asynchrony of parallel execution paradigms used in the underlying algorithms.

To be more specific, ABySS was used to assemble the genomes with a single 6-core AMD Opteron™ processor (2.1 GHz) and 128 GB of RAM [99]. The amount of computation time to generate the assembly was 300 h. In the white paper for CLC assembly Cell 4.0 software, it presents the performance of CLC *de novo* assembler on the NGS dataset of a HapMap sample, NA18507 [100]. To obtain 44× coverage, an Illumina GAII platform was used to generate paired-end reads with an average insert size of 300 bases and average read length of 101 bases. When a machine with two Intel Xeon X5550 CPUs (total of 16 cores with 2.66 GHz) was used, it took 11 h and 49 min to assemble an individual’s genome with a peak memory usage of 45 GBs. Using a different configuration with four CPUs of Xeon E7-4870 (total of 80 cores with 2.40 GHz) reduced the total assembly time approximately to 7 h [101].

These results demonstrate the demanding nature of the *de novo* genome assembly process. Similarly, experiments by authors of this publication demonstrated that running Velvet, Abyss, FALCON under different memory configurations (24 to 512 GB RAM) and number of parallel threads (16 CPU cores to 64 cores) lead to vastly different outcomes. For example, authors have performed *de novo* assembly of one of the simplest self-sustaining bacteria (~837 kbases) *Mycoplasma hiorhinis*, which has tandem repeats, inversions, complete and partially degraded potentially self-overlapping copy number and structural variants. The situation is so drastically unreliable that the assembly under different memory configurations created contigs, which align to the original known genome and to each other in only ~40%–70% of the contig’s frame. Some of the assemblies were 15% longer or shorter compared to an experimentally known construct [102]. Similar agnostic and “all-in-one” attempts to assemble personalized human genome in a single run, which is about 4000 times more complex size-wise and ~16 million times more complex computationally, needs significantly more sophisticated and careful consideration before trusting the results from any assembler.

### 6.3. Cloud Computing

Cloud computing is the most recent and promising computing paradigm. It can be described as “computation-as-a-service” and has already started to help researchers to efficiently analyze the increasing amounts of NGS data for many purposes, such as identifying SNVs, CNVs, and the *de novo* genome assembly, *etc.* Instead of purchasing the hardware for high-performance computing required for different genomics analysis purposes, this paradigm suggests the temporary rental of these services from a service provider such as Amazon Web Services (AWS), with its Elastic Cloud Computing (EC2) service, Google, and Microsoft [103,104,105]. There are three widely accepted cloud service models:

(1) *Infrastructure as a service (IaaS)*: In this model, the service provider offers the computing infrastructure that includes computational, storage and network resources as a service. Amazon EC2, Google Compute Engine and Microsoft’s Azure cloud services are the examples for this model type. Users, however, should be aware of significant costs associated in development and adaptation of tools, moving data to and from the environment. The Cloud does not provide end to end solutions; it does provide computers and network hardware with some job scheduling and system deployment facilities, most of which are usually not attuned to bioinformatics and big data I/O heavy processes.
(2) *Platform as a service (PaaS)*: The provider gives the freedom to the users to run their applications on the cloud using the provided computing platforms, which typically includes operating system, programming language execution environment, database, web servers, *etc.* The service provider hides the implementation details from the users. For this model, Amazon’s EC2 and Windows Azure can still be considered as primary examples as they provide both IaaS and PaaS services to users.
  Theoretically, all the *de novo* genome assembly methods designed to work in a parallel fashion on a HPC, can also be utilized to work on a cloud computing environment, when the underlying cloud platform or the infrastructure is set-up as a HPC. However, efficient methods are needed to distribute the computation across multiple nodes in a HPC or cloud computing environment. For this purpose, methods, such Contrail and CloudBrush, are specifically designed to use Hadoop, an open source implementation of the MapReduce that was developed by Google to simplify large data processing needs by scaling computation across many computers [106,107,108].
  The challenge in this approach is the limited efficiency in the generic environments supported by cloud providers. Small to medium size compute units available are usually affordable, however, the *de novo* assembly, being memory intensive requires larger memory, and more CPU configurations which costs significantly more. Extremely I/O and memory heavy processes (such as assemblers) encounter additional difficulties in moving data from one compute unit to another for parallel execution. Message Passing Interface (MPI) [109], shared memory or other message communication paradigms can be very challenging in cloud environments working through generic network configurations which are optimized for running small internet stores, but are not optimized for heavy tasks with significant reliance on message passing.
(3) *Software as a service (SaaS)*: The provider supplies all the software and databases to the user as a service, which eliminates the need to install and maintain the software. To mention a few: Illumina’s BaseSpace service with storage, read mapping, variant calling and *de novo* genome assembly services (backed by AWS) [110]; DNANexus’s cloud service (also backed by AWS) with tools for ChiP-seq, RNA-seq, read mapping, and variant detection [111]; High-performance Integrated Virtual Environment (HIVE) with storage and various tools including reference based or *de novo* assembly services hosted on enterprise, appliance or cloud deployments [112]; Galaxy providing spectrum of miscellaneous tools for mapping and *de novo* assembly through cloud or datacenters can be considered as major examples [113]. Another commercial example of SaaS is Life Technologies’ LifeScope cloud computing service, which provides one core of a 2.4-GHz Xeon processor with a 4 GB memory for $0.17 per core hour. There are also open source cloud solutions such as BioKepler, GenomeSpace, and Cloud BioLinux, which are accessible through Amazon EC2 cloud, as well as downloadable versions [113,114,115].

Theoretically, all the *de novo* genome assembly methods designed to work in a parallel fashion on an HPC, can also be utilized to work on a cloud computing environment, when the underlying cloud platform or the infrastructure is set-up as an HPC. However, efficient methods are needed to distribute the computation across multiple nodes in an HPC or cloud computing environment. For this purpose, methods, such as Contrail and CloudBrush, are specifically designed to use Hadoop, an open source implementation of the MapReduce that was developed by Google to simplify their large data processing needs by scaling computation across many computers [106,107,108].

The challenge in this approach is the limited efficiency in the generic environments supported by cloud providers. Small to medium size compute units available are usually affordable; however, the *de novo* assembly, being memory hungry requires larger memory, and more CPU configurations that cost significantly more. Extremely I/O and memory heavy processes (such as assemblers) encounter additional difficulties in moving data from one compute unit to another for parallel execution. Message Passing Interface (MPI) [109], shared memory or other message communication paradigms can be very challenging in cloud environments working through generic network configurations that are optimized for running a small internet stores but are not optimized for heavy tasks with significant reliance to message passing.

## 7. Quality Metrics and Parameters for Assembled Genome

As the “ground” truth of genome sequence for the individual subjected to WGS is unavailable, it is important to establish quality metrics and parameters in order to evaluate the validity of assembled genome. By looking at the measurements of the continuity, completeness, and accuracy of an assembled genome, we can estimate the quality of assembly outcomes. Gurevich *et al.* developed a quality assessment tool for genome assemblies (QUAST) with over two dozen quality parameters [116]. Even though the tool was tested on small genomes, parameters such as contig sizes, misassemblies and structural variations, genome representation and functional elements are suitable for the human genome.

Specifically, N50 is a widely used parameter to measure success of genome assembly by looking at the length of contig. It is the length for which the sum of all contigs of that length or longer is greater or equal to 50% of assembled genome. This metric only uses a single point and thus cannot accurately reflect the completeness of assembled genome. A graph plot based on continuous percentage points (1%–100%) will provide a better view of contig lengths in any series and thus direct comparison when lines of multiple assemblies are plotted in one graph [73].

In addition, assembled contigs will be used to map against the target genome to determine the completeness of assembled results. For human genome assembly, the assembled contigs can be mapped sequentially to the reference genome, alternative assembled genome, chimp genome, and genomes from human pathogens. Mapping statistics from each alignment could provide information as to its completeness, accuracy, as well as the status of infection of human pathogens in tested individuals. Mapping statistics from these four references could provide a matrix for the completeness and accuracy of genome assembly.

The tool to COMPare a DNA sequence ASSembly to trusted reference sequence (COMPASS) provides statistics and graph plots for coverage, validity, multiplicity, and parsimony [117]. The coverage is the fraction of the reference genome that was covered by assembled contigs. The validity is the fraction of the alignable region to the total length of assembled sequence. The multiplicity is the ratio of the length of alignable assembled sequences to the total covered reference genome. The parsimony is an indicator for how many bases of the assembled sequence might have errors.

REAPR is a pipeline which uses sequence reads mapping against assembled contigs, assesses the assembly quality and breaks contigs in which error is discovered [118]. Using fragment coverage distribution (FCD), REAPR generates plots and determines possible errors within assembled contigs. Most local errors detected by REAPR would be structural errors due to repetitive sequences that would complicate joining of contigs during assembly process.

In summary, recommended parameters for quality assessment of assembled genome are listed in Table 4. All tools mentioned above have been tested only on small genome assemblies or simulated data sets. Performance and validation on full human genome assemblies need to be carried out with a comprehensive study design and analysis. As human genome is more complex in content and much larger in size, further development of these tools are expected in order to guarantee good performance.

## 8. Perspectives and Remaining Challenges

Since the first whole human genome sequence completion, the reference genome has been the anchor of genome science; in reference-based studies of mapped sequences, all types of genetic variants, indels (insertions and deletions) and gene fusions were detected ; whereas, unmapped reads are generally ignored.

A lack of diploid information is another big pitfall of the reference genome. For example, there would be more mapping bias on these sites with non-reference alleles, since these alleles, when detected, were considered as mismatches and added to the mapping criteria threshold. In addition, the increase of reference and non-reference alleles would bring more false positive SNV calls. Secondly, individual copy number variation of chromosome segments, as well as short tandem repeats (STRs) might not be accurately detected by the reference genome. Furthermore, indels detected on the reference genome would lead to more unmapped reads, similar to the reference genome construction. Studies have revealed that *de novo* assembly could generate multiple novel contigs and many of them could be mapped to alternative references [119], indicating current reference genome is still far from complete. Therefore, it is the time to leverage rapid developing of sequencing platforms, assembly approaches and computer science, to improve the genome reference for better assembly.

Currently, there are two major directions for assembly improvement. The first is to further improve the reference genome, such as using a population reference graph [120]. The idea of a population reference graph is to annotate as much as possible variations on a current personal genome, in order to reduce the chance of unmapped reads. The genome would look like a network graph rather than a linear representation. In other words, a genome would be represented in an improved and alternative format as an assembly based on multiple reference genome branching in localities to a particular heterogeneous sub-population characteristic sub-sequence and then collapsing back to a combined consensus in conservative regions [120]. The challenge for detecting genomic heterogeneity is the time and computational cost of mature graph genome reference assembly. Another challenge of generating personal genome is the diploid nature of human genome, which needs to be carefully considered during the assembly of so called haplotype-resolved genomes. We expect that using diploid genome would significantly reduce the unmapped reads or contigs and overcome the mapping bias on alleles, germline mutations and overall reliability of variant and in-del calling. For example, Cao *et al.* used fosmid and NGS technologies to generate the first haplotype-resolved genome sequences by *de novo* assembly [121]. A significant number of SNPs and indels identified by *de novo* assembly were novel and not present in the current knowledge base. This indicates that using *de novo* assembly is certainly capable of finding novel genomic information, which is not represented by using the reference genome. However, the cost and time for generating this haplotype-resolved genome in the study was too high to be practical in clinical settings. Nevertheless, more *de novo* assembly approaches for haplotype-resolved genome are expected to emerge in future. Technologies such as chromosome separation or sorting with a microfluidic device, or droplet PCR to amplify genome fragments in large scale, are being developed [122,123,124,125,126,127,128]. Full development of these automation technologies would significantly simplify sample processing for haplotype-resolved genome sequencing.

As sequencing and its associated sample processing technologies continue evolving rapidly to facilitate human genome assembly, some key bioinformatics challenges, shown below, need to be addressed in order to establish personal genome as reference for clinical applications.

(1) *Quality metrics for personal genome assembly assessment.* Currently, there is no “gold standard” for personal genome assessment. Many parameters need to be considered in genome assembly assessment, including completeness, continuity, accuracy, *etc.* There have been many scoring metrics developed for genome assembly assessment [73,74,75]. However, not all of them have been directly applied for human genome assessment.
(2) *Best practice of personal genome assembly workflow.* As stated in Section 4 (Current solutions for *de novo* assembly and post-assembly), there is no single “one-size-fits-all” pipeline for *de novo* genome assembly. However, this conclusion was made based on studies of using simulated data or a single chromosome of the human genome [72,75]. Therefore, a comprehensive study needs to be performed on human genome assembly with real sequencing data. The best practice guidelines for a personal genome assembly pipeline starting from study design and ending with bioinformatics data analysis will be derived from such a study and will allow understanding of the relationships among various parameters such as NGS platforms, read length, sequence coverage and assembly process.
(3) *Personalized genome annotation.* In order to use a personal genome as reference to uncover genetic variations in diseased tissues, each individual genome needs to be well annotated with various biological features. A comprehensive bioinformatics process needs to be established to perform multiple tasks such as comparative genome analysis with the public reference genome (to identify SNPs/SVs and create cross-reference), structure analysis of gene/transcripts on the genome, identification of functional genomic loci, and calculation of sequence conservation score at each base position. This basic information may facilitate the understanding of biological effects for variants discovered in diseased tissue from the same individual.

For genome annotation, the most important components are the definition of gene structure, transcription loci, and regulatory regions. While this can be accomplished by cross-mapping a personal genome against the reference genome and consequently inferring functional loci, it will miss those regions that are unique to an individual. Thus, the best strategy is to predict gene structures with existing evidence such as expressed sequence tag (EST), cDNA, protein sequence and sequences from whole transcriptome sequencing (a.k.a RNA-Seq). There are a numbers of existing tools for gene prediction, such as SNAP [129], GENSCAN [130,131], GeneID [132], mGene [133], BRAKER1 [134], and AUGUSTUS [135,136]. However, installation and running of these tools are not trivial for general users. Web-based implementation, such as WebAUGUSTUS [137], and mGene.web [138], would provide user friendly interfaces for running gene prediction. Nevertheless, even such an implementation would not change the fact that a full run of annotation on a human genome could take weeks [139]. Therefore, another challenging task for building personal genomes is to establish a good framework for an intuitive, efficient, and less computing intensive gene annotation process that can run on a workstation.

## 9. Application in Pharmaceuticals and Pharmacogenomics

Better understanding personal genome sequence as well as the sequences of pathogenic organisms should provide valuable insights for design of targeted therapeutic agents (pharmaceuticals), and to perform tailored drug therapy in patients (pharmacogenomics). DNA sequence can also provide information for target selection, rational drug design, and genome-based stratification of patients to achieve higher efficacy and lower adverse drug reaction rate by using genotype-specific drug prescriptions [14,140,141,142].

The “genomic era” has dramatically changed the fundamental approaches in vaccine development for infectious disease. Traditionally, vaccine design is an empirical process that is time consuming and has many limitations on the success. It is particularly very difficult to develop a highly effective vaccine when the antigen is highly diverse (such as the human immunodeficiency virus); the virus is constantly mutated (such as the influenza virus); it is difficult for the virus to be cultivated in the laboratory (such human hepatitis C virus); the bacteria cannot infect animal models (such as Neisseria); or the virus has complex mechanisms in pathogenesis (such as retrovirus). Emerging with next-generation sequencing technologies, genomic-based approaches of vaccine development may significantly increase the effectiveness, efficacy and specificity of vaccines. As summarized by Seib *et al.* [141], the genomics-based approaches, incorporating omics knowledge and strategies for vaccine development include: 1) metagenomics data to identify causative pathogen of a disease; 2) using genomic, transcriptomic, proteomic information of the pathogen to identify targets on the pathogen for vaccine development; 3) comparing the genome of pathogens with the personal genome information to avoid choosing the identical, similar or homologous regions between host and pathogen genomes for the vaccination targets; 4) validation of candidate vaccines by *in vivo*, *in vitro* and clinical studies based on genomic information derived from the pathogen and patients to show a high clinical efficacy and a low adverse reaction rate; and 5) vaccine approval and licensing. Similar approaches are suitable for the development of antibiotics. To date, more than 20,000 metagenomics projects with many terabytes of sequencing data have been produced and they are publicly available [143]. Therefore, the sequence information from both the hosts and pathogens is a rich resource for the identification of therapeutic targets, and for the design, development and validation of the therapeutics (vaccines and antibiotics).

Genetic variants in genes encoding DMETs have been linked with inter-individual variability in drug efficacy and safety. Much clinical evidence has demonstrated the success of using genetic markers to predict drug efficacy and safety [14]. The practice of precision medicine and pharmacogenomics requires the identification/qualification of biomarkers to distinguish individuals by their differences in genetic make-ups that are responsible for disease susceptibilities, variable responses to drug treatments or the risks of drug adverse reactions. However, the number of useful pharmacogenomics biomarkers is still relatively small, owing to the lack of convincing validations. Biomarker identification is complicated by many confounding factors, such as the complexity of the human genome, the quality of sequencing data, the genetic heterogeneity within a study population, the limitation of phenotypic/genotypic sample size, and the variety of pharmacological mechanisms. Unfortunately, there is no “gold standard” as to what approaches would sufficiently and optimally assess genetic variants that are associated with drug efficacy or safety. To facilitate the establishment of suitable pharmacogenomics biomarkers, the FDA Biomarker Qualification Program was established to provide a framework in qualifying biomarkers [144]. As a guideline for processing the development and evaluation of biomarkers, this program provides a framework to integrate the information gathered for qualified biomarkers and encourages new biomarker identification and utilization.

Large inter-individual variability in the expression of common DMETs has been documented [145,146], which may be mainly due to the influence of genetic variants, such as single nucleotide polymorphisms and copy number variations. Over the years, studies have linked the genetic variants with the altered expression of DMETs, drug metabolisms, and drug efficacy and drug safety [14,15,16]. The FDA has created a database of genetic variants that may affect the treatment outcomes for drugs with evidence of adverse reactions [147]. However, the list of genetic variants does not represent the full spectrum of genetic variations in DMET genes and drug targeted genes. DMET genes are “difficult genes” that possess challenges in the sequencing process. First, DMET genes are highly homologous among gene family members. For example, cytochrome P450 genes being assigned to the same gene family are based on the criterion of a primary sequence homology greater than 59%, while cytochrome P450 genes are placed within the same subfamily based on the consideration of at least 70% similarity in sequences among genes [148]. Second, sequence variation that results in functional variants of DMET genes can occur across the entire genes, from 5’-flanking regions to the 3’flanking regions, including the intergenic area [149]. Some of the functional variants may be located far from the coding region and affect the gene expression [150]. Third, there are many pseudogenes, even transcribed pseudogenes, which can affect the expression of DMETs. For example, it was reported that the CYP2A7 pseudogene transcript may increase the CYP2A6 protein production [151]. In order to overcome these challenges to correctly survey the genetic variations of DMET genes, precise sequencing platforms with appropriate sequencing depth and base-calling precision, better assembly algorithms for pseudogene removal and specifically mapping homologous regions to counterpart genes are warranted.

HLA gene mediated drug adverse reactions, such as HLA-B*57:01-associated abacavir-induced hypersensitivity [152] and HLA-A*31:01-associated carbamazepine-induced skin injury [153], are unique pharmacogenomics cases by which the drug adverse reaction associated genetic variants are located in neither the DMET genes nor the drug targeted genes. However, the resolution of HLA genotyping is very fuzzy. The HLA genes, including the class I cluster (HLA-A, HLA-B, and HLA-C) and class II cluster (HLADP, HLA-DM, HLA-DOA, HLA-DOB, HLA-DQ and HLA-DR), are the most polymorphic genes in the human genome, with a huge number (hundred thousands) of possible variants by the dimerization of a class I molecule and a class II molecule that results in extensive allelic diversity [154]. The large number of polymorphisms in the gene, the high complexity of the dimerized molecule, the linkage disequilibrium, and the clustered nature of extensive allelic diversity make the high-resolution HLA genotyping a great challenge [14]. We expect that the advances of next generation sequencing technologies, more depths of sequencing coverage, longer reads of the sequencing reaction, higher accuracy of the base calling, better assembly pipelines, more precise haplotype construction algorithms and simplified workflow will facilitate the accuracy and the throughput of HLA genotyping.

As most of the linkages between genotype and phenotype were established through technologies that detect SNP alleles separately, the phasing information of SNP sites within a gene are completely lacking. However, the combination of multiple genetic variant loci may provide the most accurate depiction of drug responsiveness among individuals. Thus, a haplotype resolved personal genome would provide a complete picture of genetic variants on both alleles and elevate the level of our understanding of genetic variants in association with drug adverse reaction.

## 10. Summary

**Figure 2:**
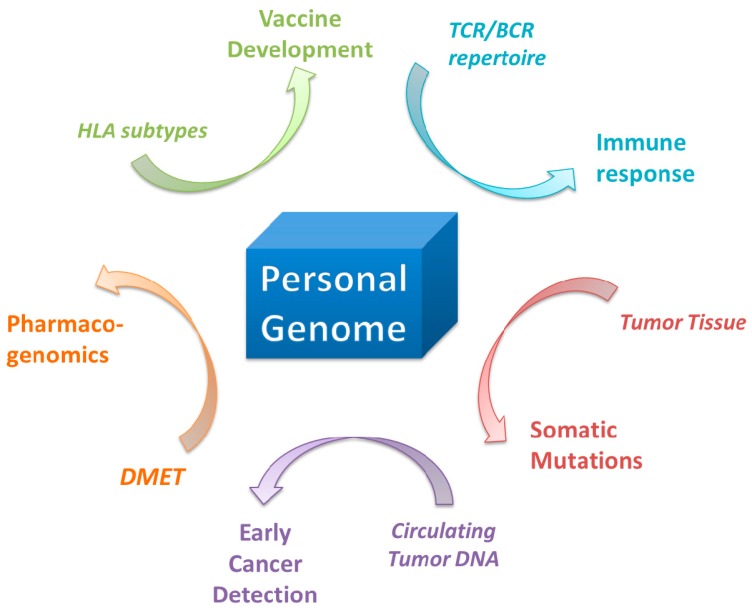
Potential use of a personal genome in future clinical settings.

The usage of a personal genome instead of a public genome as reference may lead to a milestone in personalized medicine and has the potential to aid in a variety of biomedicine realms such as infectious diseases, pharmacogenomics and tumor detection and therapeutics. An accurate and well-annotated personal genome is a pivotal base for *N*-*of-1* trials. With an established personal genome as a reference, somatic mutations in the tumor biopies would be more readily detected. Deep sequencing of circulating cell free DNA (ccfDNA) from regular blood draws would enable early cancer detection. TCR/BCR repertoire profiling would allow us to monitor host immune-response when treatment was given. A complete understanding of genetic variants in HLA and DMET genes on both haplotype alleles would give us power in accurately predicting drug adverse effects or designing personalized vaccines (Figure 2).

Overall, for human genome sequencing technology, there are trade-offs between cost and accuracy, between read-length and high-throughput, and between time and read-depth. With the continuous development of sequencing platforms, sample processing technologies, and assembly approaches, it may not be long before personal genome with high quality diploid information will be available with affordable costs.

There are many big challenges remaining to establish the best practice for genome assembly and annotation. Due to the scope of challenges, lengthy time in running each *de novo* genome assembly and complexity in post-assembly analysis, a consortium effort, like the Assemblathon, is needed in order to advance personal genome assembly.

## Figures and Tables

**Table 1:** Basic statistics for the recent releases of human reference genome build.

Genome Build #	Release Year	Total Genome Length	Total Non-N Bases	N50	Number of Gaps	# of Scaffolds	# Unplaced Scaffolds
35	2004	3,091,649,889	2,866,200,199	38,509,590	292	377	86
36	2006	3,104,054,490	2,881,649,121	38,509,590	292	367	88
37	2009	3,137,144,693	2,897,299,566	46,395,641	357	249	59
38	2013	3,209,286,105	3,049,316,098	67,794,873	875	473	169

**Table 2:** Comparison of current common NGS platforms.

Platform	Mode	Read-Length	Reads Passing Filter per Run	Output	Run Time	Quality	Cost/Run	Instrument Price
Illumina HiSeq 2000/2500	High-Output	1 × 36–2 × 125	4 B	128 GB–1 TB	1–6 days	Q30 ≥ 80%	~$29K	$740K
	Rapid	1 × 36–2 × 150	600 M	18 GB–300 GB	7–60 h	Q30 ≥ 75%	~$8K	
Illumina HiSeq X ten	X ten	2×150	5.3–6 B	1.6–1.8 TB	<3 days	Q30 ≥ 75%	~$12K	$1M*
Roche 454 FLX system	Titanium XL+	700	1 M	700 MB	23 h	99.997%	~$6K	~$500K
Life Technologies Ion Torrent	Proton I	200	165 M	~10 GB	2–4 h		~$1000	$149K
	Proton II	100	660 M	~32 GB	2–4 h			
Intelligent Biosystems (Qiagen)	MAX-Seq	2 × 55	75 M/lane	132 GB	2.5 days		~$1200	~$270K
	Mini-20	2 × 100	20 M/lane	80 GB			~$150–300/sample	$125K
PacBio RS	RS II	10–15 KB	50 K	500 MB–1 GB	4 h	>99.999%	~$400	~$700K
Oxford Nanopore	miniON	>200 KB	no fixed run time (~1 bp per nanosecond)				≤$900	~$1000

* K: thousand; M: million; B: billion; kb: kilobase; MB: millionbase; GB: gigabase; TB: terabase; h: hour.

**Table 3:** Genome *de novo* assembly and post-assembly approaches.

Approaches	Commonly Used Tools	Notes
**Assembly Approaches**		
*de Bruijn* graph	EULER, ALLPATHS, Velvet, ABySS, SOAPdenovo, *etc.*	For shorter reads (25–100 bp) assembly
Overlap-layout-consensus (OLC)	SSAKE, SHARCGS, VCAKE, Celera Assembler, Arachne, PCAP, HGAP, *etc.*	For longer reads (100–800 bp) and long reads assembly
**Post-Assembly Approaches**		
Contigs orientation and visualization	AlignGraph, ABACAS, CONTIGuator, Projector2, OSLay and r2cat, *etc.*	
Extending contigs and filling gaps	IMAGE, GAA program, Reconciliator, GAPFiller, Pilon *etc.*	
Reads error correction	ICORN, AutoEditor, REAPR *etc.*	
Unmapped reads Annotation	RATT, Ensembl, GARSA and SABIA, *etc.*	

**Table 4:** Parameters for quality assessment of assembled genome.

Parameters		Notes
**Contig Statistics**		
Number of contigs		total number of assembled contigs
Max length of contigs		the longest contig
Min length of contigs		the shortest of contig
Total length of contigs		sum of the length of all contigs
Nx_plot		contig length for x% of the bases of assembled contigs, where 0 < x < 100
NGx_plot		contig length for x% of the bases of reference genome, where 0 < x < 100
NAx_plot		contig length for x% of the bases of assembled contigs after correction, where 0 < x < 100
NGAx_plot		contig length for x% of the bases of the reference genome after correction, where 0 < x < 100
**Assembly Errors**		
Number of misassembles		total number of assembly errors, include miss-join, base error, false indel, *etc.*
	miss-join	number of miss-join
	base error	number of base error
	false indel	number of false indel
Number of misassembled contigs (parsimony)		number of contigs with assembly errors
Total length of misassembled contigs		sum of the length of misassembled contigs
Unaligned cotigs		total number of contigs could not be mapped to the reference genome
	alternative human reference	could be mapped to alternative human reference genomes
	nonhuman primate genome references	could be mapped to nonhuman primate reference genomes
Ambiguously mapped contigs		cotigs mapped to multiple location on the reference genome
Fragment coverage distribution (FCD)		local assembly error detected by fragment coverage of assembled contigs by sequence reads
**Genome Coverage**		
Genome coverage fraction		percentage of the reference genome covered by assemblies
Known gene complete coverage fraction		percentage of known gene covered completely by assemblies
Known gene partial coverage fraction		percentage of known gene covered partially by assemblies
Know exon complete coverage fraction		percentage of known exon covered completely by assemblies
Know exon partial coverage fraction		percentage of known exon covered partially by assemblies
Duplication ratio (multiplicity)		ratio of total length of aligned contigs *vs.* total covered the reference genome
Alignable ratio (validity)		ratio of total aligned contigs *vs.* total assembled contigs
GC content		percentage of GC content in assembled contigs
Number of SNVs		total number of single nucleotide variation (SNV) detected in assembled contigs
Number of SNPs		total number of single nucleotide polymorphism (SNP) detected in assembled contigs
Number of small indels		total number of small indels detected in assembled contigs
Number of inversion		total number of inversion detected in assembled contigs
Number of translocation		total number of translocation detected in assembled contigs
SNVs/100 kb		number of SNVs per 100 kb block
SNPs/100 kb		number of SNPs per 100 kb block
indels/100 kb		number of small indels per 100 kb block
